# Cardioembolic Sources in Stroke Patients in South of Brazil

**DOI:** 10.1155/2014/753780

**Published:** 2014-10-02

**Authors:** Luiz Carlos Porcello Marrone, João Pedro Farina Brunelli, Ricardo Lutzky Saute, Gustavo Henrique Tomasi, Bianca Cecchele Madeira, William Alves Martins, Robson Dupont Rohr, Ana Paula Heck, Luiz Ricardo Botton, Marilia Martins de Castro, Rodrigo Bodanese, Luiz Carlos Bodanese, Antônio Carlos Huf Marrone, Jaderson Costa da Costa

**Affiliations:** Hospital São Lucas, Instituto do Cérebro, Pontifícia Universidade Católica do Rio Grande do Sul, Avenida Ipiranga 6690, Room 220, 90610-000 Porto Alegre, RS, Brazil

## Abstract

*Background.* Stroke is a leading cause of mortality and disability in Brazil and around the world. Cardioembolism is responsible for nearly 30% of the origins of ischemic stroke. *Methods.* We analyzed data of 256 patients with cardioembolic ischemic stroke (according to TOAST classification) who were admitted into the Hospital São Lucas-PUCRS from October 2011 to January 2014. The cardioembolic subtype was divided into six subgroups: arrhythmias, valvular heart disease, coronary artery disease, cardiomyopathy, septal abnormalities, and intracardiac injuries. The prevalence of the most important cardiovascular risk factors and medications in use for prevention of systemic embolism by the time of hospital admission was analyzed in each patient. *Results.* Among 256 patients aged 60.2 +/− 6.9 years, 132 males, arrhythmias were the most common cause of cardioembolism corresponding to 50.7%, followed by valvular heart disease (17.5%) and coronary artery disease (16%). Hypertension (61.7%) and dyslipidemia (43.7%) were the most common risk factors. Less than 50% of patients with arrhythmias were using oral anticoagulants. *Conclusions.* Identifying the prevalence of cardioembolic stroke sources subgroups has become an increasingly important role since the introduction of new oral anticoagulants. In this study, arrhythmias (especially atrial fibrillation) were the main cause of cardioembolism.

## 1. Introduction

Stroke is the leading cause of mortality and disability in Brazil and South America. However, there is little knowledge about stroke epidemiology, stroke subtypes, and risk factors in Latin America [1–3].

Basically strokes can be divided into ischemic (85%) and hemorrhagic (15%). The clinical characteristics of stroke vary according to etiology and risk factors. To facilitate and standardize the classification of stroke subtypes, it was developed in 1993—the TOAST (Trial of Org 10172 in Acute Stroke Treatment) classification which divides the ischemic stroke into five subtypes: large-artery atherosclerosis, cardioembolism, small-vessel occlusion, stroke of other determined etiology, and stroke of undetermined etiology. Knowledge of the etiology of ischemic events is essential for correct treatment and secondary prevention to improve the best patient outcomes [4, 5].

In the south of Brazil, the most important source of ischemic stroke is large-artery atherosclerosis followed by cardioembolism and small-vessel disease. Cardioembolism is responsible for nearly 30% of the origins of ischemic stroke [2, 6, 7]. Cardioembolism can be subdivided into six subgroups: arrhythmias (as atrial fibrillation (AF) and atrial flutter), valvular heart disease, cardiomyopathy, coronary artery disease (within six weeks), septal abnormalities, and intracardiac injuries [1, 6, 7].

Currently, new therapies are being introduced and these are specific to certain cardioembolic diseases. Nowadays, new oral anticoagulants (NOAC) have been used to better control nonvalvular atrial fibrillation [8]. Because of this, it is important to know more specifically which subgroups of cardioembolic stroke are responsible for more cases in our population.

The objective of this paper is to describe the most common causes of cardioembolic stroke and its risk factors.

## 2. Materials and Methods

We reviewed data from 256 consecutive patients with acute cardioembolic ischemic stroke admitted to Hospital São Lucas in Porto Alegre (Brazil) between October 2011 and January 2014. Porto Alegre is a city in southern Brazil with a population of 1.4 million inhabitants, predominantly of European and African origin. We analyzed the prevalence of subgroups of cardioembolic stroke, which was defined according to TOAST.

Our study was carried out exclusively with patients admitted to the hospital's emergency department. In this investigation, all patients were submitted to brain computed tomography and/or magnetic resonance imaging, electrocardiography, chest radiography, and basic blood tests. The majority of patients also underwent transesophageal echocardiography, Doppler ultrasonography of the neck vessels, and/or magnetic resonance angiography.

We used TOAST classification to define the cardioembolic subgroup. The cardioembolic stroke was then divided into six subgroups: arrhythmias (like atrial fibrillation and atrial flutter), valvular heart disease, coronary artery disease, cardiomyopathy, septal abnormalities, and intracardiac injuries. We evaluated the following variables in each group: age, sex, hypertension, diabetes, dyslipidemia, smoking, and medications used for prevention of systemic embolism by the time of hospital admission.

Hypertension was defined on treatment of antihypertensive therapy or 2 measurements of systolic pressure >140 mm Hg and/or diastolic pressure >90 mm Hg. Diabetes mellitus was defined as a history of elevated serum glucose level (>100 mg/dL) on 2 independent readings before stroke or receipt of antidiabetic medications. Dyslipidemia was defined as a history of total cholesterol >200 mg/dL or triglycerides >200 mg/dL or receipt of lipid-lowering medications.

Patients who presented in the emergency department with previous stroke (without acute lesions) or who had been treated as outpatients were not included in the sample. The stroke subtype was identified after review of all imaging data. All statistical analyses were performed using SPSS version 13.0 (SPSS Inc., Chicago, IL).

According to TOAST classification, subgroups of cardioembolism could be divided into high and medium risk for stroke. The high risk includes mechanical prosthetic valve, mitral stenosis with AF, AF other than lone AF, left atrial/atrial appendage thrombus, sick sinus syndrome, recent myocardial infarction (<4 weeks), left ventricular thrombus, dilated cardiomyopathy, akinetic left ventricular segment, atrial myxoma, and infective endocarditis. The medium risk includes mitral valve prolapsed, mitral annulus calcification, mitral stenosis without AF, left atrial turbulence (smoke), atrial septal aneurysm, patent foramen ovale, atrial flutter, lone AF, bioprosthetic cardiac valve, nonbacterial thrombotic endocarditis, congestive heart failure, hypokinetic left ventricular segment, and myocardial infarction >4 weeks and <6 months [4, 9].

## 3. Results

We analyzed data from 256 patients with mean age of 60.2 ± 6.9 years, 132 males (51.5%) and 124 females (48.4%).

In our series, arrhythmias were the most common cause of cardioembolism, followed by valvular heart disease and coronary artery disease. The arrhythmias subgroup was composed of 130 patients with mean age of 58.1 years and 96.1% of these patients had atrial fibrillation as the cause of ischemic stroke. Hypertension (61.7%) and dyslipidemia (43.7%) were the most common risk factors in all subgroups.

Table 1 shows the prevalence of each subgroup of cardioembolism and risk factors.

Evaluating 256 patients with cardioembolic subtype, only 120 (46.8%) were using anticoagulants for prevention of systemic embolism.  Table 2 depicts the medications for prevention of systemic embolism that the patients were using by the time of their admission to our hospital.

## 4. Discussion

Cardioembolic stroke predicts long-term mortality when compared to other ischemic stroke subtypes [10]. Moreover, this condition had great rates of reperfusion in patients treated with rt-PA; however hemorrhagic transformation is more common [11, 12]. The cardioembolic stroke is also associates with a large in-hospital mortality and worse prognosis [1].

In a previous paper of our group, we found that cardioembolism was the cause of ischemic stroke in 28.3% of the patients. Porcello Marrone et al. and Ihle-Hansen and colleagues found a very similar prevalence of cardioembolism of 31.4% analyzing 210 patients with ischemic stroke [1, 6].

In Spain, Capmany et al. described a prevalence of 20% of cardioembolic stroke. Furthermore, in this series 79.1% of patients had AF with or without structural cardiac disease associated with arrhythmias. The main correlation was found with AF and hypertensive left ventricular hypertrophy; this finding is similar to that found in the present study. In our study, the prevalence of valvular heart disease was 17.5%, whereas in the Spanish study it was 12%. The same occurred in relation to coronary artery disease, where we found 16% and they found 20% [13]. Maybe what explains our high number of valvular heart disease is the increased prevalence of rheumatic fever in our midst [14].

Chagas disease is described as an important cause of cardioembolic stroke in some regions of Brazil. However, in Porto Alegre this disorder is not as common as in the central regions of Brazil and South America [15–17].

In our series, less than half of the patients with AF that arrived in our hospital due to a stroke were using anticoagulants.

Identifying the prevalence of cardioembolic stroke sources subgroups has become increasingly important since the introduction of NOAC for specific diseases related to that situation. Novel therapies—dabigatran (direct inhibitor of prothrombin) [8], rivaroxaban, and apixaban (direct Xa inhibitor) [18, 19]—proved to be at least as effective as warfarin to prevent stroke in patients with AF. Evaluating only dabigatran 150 mg twice a day, this drug proved to be superior in ischemic stroke prevention when compared to warfarin [8].

We know that this paper has some limitations, but some information described here can help neurologists and clinicians with their practice.

In summary, nearly 50% of cardioembolic stroke patients admitted to Hospital São Lucas-PUCRS are due to arrhythmias. Thus, a greater number of patients can make use of those NOAC once that only half of the patients who could benefit from anticoagulation are actually being treated, probably due to the poor safety profile of Warfarin [8]. Possibly, the number of strokes caused by atrial fibrillation is larger than that described in this paper, due to paroxysmal atrial fibrillation cases that may not have been diagnosed.

## Figures and Tables

**Table 1:** Cardioembolic sources and risk factors.

Cardioembolic subtypes	*n*	%	Age	Male (%)	Hypertension (%)	Diabetes (%)	Smoking (%)	Dyslipidemia (%)
Arrhythmias	130	50.7	58.1	60 (46.1)	69 (53)	38 (29.2)	43 (33)	55 (42.3)
Valvular heart disease	45	17.5	61.2	27 (60)	31 (68.8)	11 (24.4)	19 (42.2)	19 (42.2)
Coronary artery disease	41	16	69.7	21 (51.2)	38 (92.6)	16 (39)	18 (43.9)	21 (51.2)
Cardiomyopathy	12	4.6	66.6	8 (66.6)	6 (50)	3 (25)	4 (33.3)	5 (41.6)
Septal abnormalities	24	10	53	14 (58.3)	11 (45.8)	6 (25)	8 (33.3)	10 (41.6)
Intracardiac injuries	4	1.5	44.2	2 (50)	3 (75)	1 (25)	2 (50)	2 (50)

Total	256	100	60.2	132 (51.5)	158 (61.7)	75 (29.2)	94 (36.7)	112 (43.7)

**Table 2:** Prevention therapy for cardioembolic stroke in hospital admission.

Cardioembolic subtypes	*n*	Oral anticoagulants (%)	Antiaggregants (%)	None (%)
Arrhythmias	130	63 (48.4)	40 (30.7)	27 (20.7)
Valvular heart disease	45	37 (82.2)	6 (13.3)	2 (4.4)
Coronary artery disease	41	11 (26.8)	27 (65.8)	3 (7.3)
Cardiomyopathy	12	5 (41.6)	6 (50)	1 (8.3)
Septal abnormalities	24	4 (16.6)	16 (66.6)	4 (16.6)
Intracardiac injuries	4	0	1 (25)	3 (75)

Total	256	120 (46.8)	96 (37.5)	40 (15.6)

## References

[1] Porcello Marrone LC, Diogo LP, de Oliveira FM (2013). Risk factors among stroke subtypes in Brazil. *Journal of Stroke and Cerebrovascular Diseases*.

[2] de Carvalho JJF, Alves MB, Viana GÁA (2011). Stroke epidemiology, patterns of management, and outcomes in Fortaleza, Brazil: a hospital-based multicenter prospective study. *Stroke*.

[3] Saposnik G, Del Brutto OH (2003). Stroke in South America: a systematic review of incidence, prevalence, and stroke subtypes. *Stroke*.

[4] Adams HP, Bendixen BH, Kappelle LJ (1993). Classification of subtype of acute ischemic stroke: definitions for use in a multicenter clinical trial. *Stroke*.

[5] O’Donnell MJ, Denis X, Liu L (2010). Risk factors for ischaemic and intracerebral haemorrhagic stroke in 22 countries (the INTERSTROKE study): a case-control study. *The Lancet*.

[6] Ihle-Hansen H, Thommessen B, Wyller TB, Engedal K, Fure B (2012). Risk factors for and incidence of subtypes of ischemic stroke. *Functional Neurology*.

[7] Caplan LR (2009). *Caplan's Stroke: A Clinical Approach*.

[8] Connolly SJ, Ezekowitz MD, Yusuf S (2009). Dabigatran versus warfarin in patients with atrial fibrillation. *New England Journal of Medicine*.

[9] Amarenco P, Bogousslavsky J, Caplan LR, Donnan GA, Hennerici MG (2009). Classification of stroke subtypes. *Cerebrovascular Diseases*.

[10] Stead LG, Gilmore RM, Bellolio MF (2011). Cardioembolic but not other stroke subtypes predict mortality independent of stroke severity at presentation. *Stroke Research and Treatment*.

[11] Ferro JM (2003). Cardioembolic stroke: an update. *The Lancet Neurology*.

[12] Molina CA, Montaner J, Abilleira S (2001). Timing of spontaneous recanalization and risk of hemorrhagic transformation in acute cardioembolic stroke. *Stroke*.

[13] Capmany RP, Arboix A, Casañas-Muñoz R, Anguera-Ferrando N (2004). Specific cardiac disorders in 402 consecutive patients with ischaemic cardioembolic stroke. *International Journal of Cardiology*.

[14] Bocchi EA, Guimarães G, Tarasoutshi F, Spina G, Mangini S, Bacal F (2009). Cardiomyopathy, adult valve disease and heart failure in South America. *Heart*.

[15] Dias Junior JO, da Costa Rocha MO, de Souza AC (2014). Assessment of the source of ischemic cerebrovascular events in patients with Chagas disease. *International Journal of Cardiology*.

[16] Carod-Artal FJ (2013). Policy implications of the changing epidemiology of chagas disease and stroke. *Stroke*.

[17] Carod-Artal FJ, Casanova Lanchipa JO, Cruz Ramírez LM (2014). Stroke subtypes and comorbidity among ischemic stroke patients in brasilia and cuenca: a brazilian-spanish cross-cultural study. *Journal of Stroke and Cerebrovascular Diseases*.

[18] Patel MR, Mahaffey KW, Garg J (2011). Rivaroxaban versus warfarin in nonvalvular atrial fibrillation. *New England Journal of Medicine*.

[19] Granger CB, Alexander JH, McMurray JJV (2011). Apixaban versus warfarin in patients with atrial fibrillation. *The New England Journal of Medicine*.

